# Retrodeviations of the Uterus1Read before the Obstetrical Society of Cincinnati, December 16, 1908.

**Published:** 1908-05

**Authors:** Charles L. Bonifield

**Affiliations:** Cincinnati, Ohio


### Retrodeviations of the Uterus[1]

By CHARLES L. BONIFIELD, M.D., Cincinnati, Ohio

[From *The Lancet-Clinic,* March 21, 1907]

NO other subject has received more attention from gynecologists during the last decade than the one I have selected to discuss with you this evening.

That so many operations have been devised to relieve patients of symptoms due, or supposed to be due, to retroversion or retroflexion of the uterus is proof positive that other methods of treatment have proven unsatisfactory, and that the operation that will achieve success when used in the indiscriminate manner of the average surgeon is yet to be perfected.

I have no new method of treatment, surgical or otherwise, to offer. I shall speak briefly of etiology, symptoms and complications to open the whole subject for discussion, and express my opinion, based on a fairly large experience, much of it with working women, of the comparative value of well-known methods of treatment.

There are three types of retrodeviated uteri:

1. The congenital. The uterus is undeveloped, and for some reason, probably less development of the posterior than the anterior wall, has assumed the attitude of sharp retroflexion instead of the more common one of anteflexion.

2. Those cases in which the deviation is neither caused nor seriously complicated by inflammation of adjacent structure.

3. Those in which the deviation is caused by inflammation, or in which the inflammatory trouble with the appendages overshadows in importance the deviation.

In the first class of cases the arrest of the development is the essential condition, and the one to which treatment should be directed. Dysmenorrhea is the symptom that is usually present and brings the patient to the gynecologist. An atrophic endometritis usually exists. A thorough dilatation of the cervix, a gentle curettage, followed by tight packing with gauze, may render the

1. Read before the Obstetrical Society of Cincinnati, December 16, 1908.

cervix more patulous, the endometrium more healthy, and thus enable the uterus to perform the menstrual function with little or no pain. It may be necessary to repeat this procedure several times, at intervals, varying from six months to two years. Not infrequently the ovaries share in the lack of development, and their removal may be necessary to free the patient from painful menstruation. Operative or other treatment to correct the retroflexion will be disappointing and should not be resorted to.

Cases of the second class occur in the young or unmarried as well as those who have borne children. In the former the cause is often obscure. The uterus is held in its normal slightly anteflexed position by its ligaments and intraabdominal pressure. The perineum cannot be considered a support, but its injury or destruction allows forces to come into play that have a tendency to displace it.

The ligaments, aside from the round ones, are little more than folds of peritoneum, whose retaining power is not great and which are at a mechanical disadvantage in maintaining the uterus in its normal place and attitude when a woman is in the upright position. If women went on all fours, as did their remote ancestors, the supports of the uterus would answer their purpose admirably well. As it is they are barely able to perform their functions under the most favorable conditions. If their task is augmented by an increase in the weight of the uterus, or if they are weakened by disease or injury, they are not equal to it. Whether it is a version or a flexion that ensues, depends on the tonicity of the uterine muscle and the strength of the sacro-uterine ligaments.

In the unmarried the weight of the uterus is increased by chronic passive congestion, the causes of which are many. Organic or functional disease of the heart is an important one, and disturbance in the portal circulation is another. Exposure during menstruation may check or arrest that function and leave the uterus without the depletion for which it has been prepared by premenstrual congestion. Vaginal injections of cold water are sometimes used to cut short a menstrual period that is interfering with business or pleasure. "Spooning" and the sexual desire it engenders causes a hyperemia of the sexual organs. Suggestive literature and plays act in the same way. Sedentary habits are also conducive to congestion. The supports of the uterus are weakened by anything that lowers the general vitality.

The effect of general vitality on intra-peritoneal conditions is well shown by the experience of a patient of mine. When about twenty-five years of age he was thought to have incipient tuberculosis. He was at least anemic and much reduced in weight. He developed double inguinal hernia, for which he wore trusses for several years. Horseback riding and diet restored him to vigorous health, and he was able to lay aside his trusses. About five years after he had discarded them a four-months' illness caused the hernias to reappear. They disappeared again when health and body weight were again restored, and have caused no trouble during the ten years that have elapsed since.

The society girl lowers her vitality by late hours, improper food and impaired digestion. The girl who works behind a counter, in a factory or sweat shop, spends so many hours a day in vitiated atmosphere that robust health is impossible. It is probable that venous congestion of the pelvic organs weakens the ligaments, thus the same causes that increase their burden lessen their strength.

Intra-abdominal pressure is reduced by lack of tonicity of the abdominal muscles, which may be brought about by lack of exercise, malnutrition, fatty degeneration, etc. The uterus may be thrown backwards by a sudden jar; a fall in which a woman alights on the buttock seems especially apt to produce this result. Horseback riding, especially by the inexperienced, may readily have the same effect. Constipation may be a cause by pushing the cervix forward. A bladder that is overdistended much of the time pushes the body of the uterus backward, so that intra-abdominal pressure is exerted on its anterior rather than the posterior surface. Tight lacing may aggravate a retrodeviation, but cannot alone produce it. In some cases retroversion seems to be the uterine contribution to general ptosis of the intra-abdominal organs.

In married women who have not been pregnant the causes enumerated for the unmarried are often responsible, but for ungratified sexual desire are substituted over-indulgence and harmful efforts to prevent conception. In child-bearing women involution is a paramount cause of a heavy uterus. Subinvolution is fully as liable to follow abortion as labor at term, and its chief cause is infection, which may be so mild as to attract little attention and soon forgotten by the attendant.

Laceration of the cervix seriously interferes with involution, and injuries to the pelvic floor cause traction on the uterus by the vagina, rectum and bladder, which tends to make the axis of the uterus conform to that of the vagina, to do which it becomes retroverted.

Resuming the upright position before involution has had time to get well under way may cause its arrest, and lying constantly on the back during the days following delivery permits the body of the uterus to gravitate backward. The ligaments have all been stretched during pregnancy, and early rising after delivery allows the uterus to be displaced.

The symptoms of posterior deviations of the uterus are those due to its increased weight, its dragging or pressure on contiguous organs, and those produced reflexly through the nervous system, including a feeling of weight in the pelvis, backache, headache, constipation and irritability of the bladder. Constipation is produced in a reflex, not a mechanical, way. A fibroid tumor growing from the posterior wall of the uterus down near the cervix may attain the size of several times that of the enlarged retroverted uterus without interfering with the passage of fecal matter through the rectum. It therefore seems impossible for the retroverted uterus, with ligaments relaxed as they are, to act in this way. In labor the pressure of the child's head against the rectum often leads the patient to think that she wishes to evacuate the bowels. The entrance of the feces into the rectum is the usual cause of the impulse to go to stool. If this impulse be habitually ignored, the pressure of the feces ceases to excite it. It is not improbable that the persistent pressure of the uterine body against the rectum obtunds its sensibility.

The irritability of the bladder is most marked in those cases in which the posterior deviation is accompanied by a noticeable descent of the uterus.

The complications of the retrodeviations of the uterus are prolapse of one or both ovaries and adhesions fastening the body of the uterus in its abnormal position. For anatomical reasons—slightly lower position and absence of valves in its veins—the left ovary is the more frequently prolapsed. The prolapsus is usually followed by cystic degeneration, and the ovary is found to be smaller than normal. No doubt it is an atrophic change in the ovary as a result of the enlargement of the veins, just as the testicle atrophies from varicocele. The ovary, being a much more sensitive organ than the uterus, its displacement causes symptoms of severer character than the retrodeviation; in fact, those attributed to the latter are at times due almost entirely to the ovary.

Adhesions which are the result of the faulty attitude of the uterus are broad and weblike, and in time become very firm, and occur in those cases in which the taut broad ligaments hold the uterus quiet and comparatively fixed in position. The uterus that flops around like a flail has no more tendency to contract adhesions than the small pedunculated fibroid of the uterus or the solid tumor of the ovary. Adhesions do not aggravate the symptoms materially unless they include an appendage, but render some of the palliative methods of treatment inapplicable.

The diagnosis of retrodeviation of the uterus is made by bimanual examination. It is not difficult, but may require an anesthetic to determine the exact condition of the appendages, which it is always important to know before deciding what line of treatment should be pursued.

The indications for treatment are to reduce the weight of the uterus and increase the strength of its supports, or provide new ones. The weight of the uterus may be reduced by rest in bed, hot douches, saline purgation, by tampons, by incising the cervix, and by trachelorrhaphy or amputation of the cervix.

The supports of the uterus may be aided by position tampons and pessaries, by shortening of the stretched ligaments, or by forming new attachments to the abdominal wall or vagina. The reduction in the weight of the uterus should be coincident with, or preferably precede, whatever operation is made to hold it in place. Sometimes a curettage is all that is required, but not infrequently in child-bearing women a trachelorrhaphy or amputation of the cervix is also necessary. In many cases where a cervical operation is not required a preliminary treatment of hot douches and tampons is very beneficial, and will materially lighten the burden the support must maintain after operation. When these operations are needed and the uterus is exceedingly large, it is better worth while to perform them than wait several weeks for involution to take place before operating on its supports. Perineal lacerations must also be repaired. Attaching the fundus to the abdominal wall gives the needed support at the point at which it works at the greatest mechanical advantage. It is easily done and gives satisfactory results even in the hands of an amateur in a large per cent. of cases, though if the attachment is made too low it may interfere with the proper distention of the bladder; if fixed too firmly it raises the uterus too high—substitutes one abnormal condition for another. If the attachment is too small the new ligaments soon become so attenuated as to be inefficient. It has caused trouble in labor in a sufficient number of cases to cause most operators to abandon its use in women in whom pregnancy is possible. It has also led to obstruction of the bowels. Personally, I think it should never be used for retrodeviations.

Vaginal fixation, devised by Mackinrodt and Duhrssen, has not given satisfaction in the hands of many. I was in Berlin in 1892 and saw both of the operators do it, but it did not appeal to me and I have never tried it. It, too, has interfered most seriously with subsequent labor. I think that it is now seldom employed, at least in this country.

The round ligaments, on account of their muscular structure permitting growth and involution and their favorable point of attachment to the uterus, are best fitted to operative treatment.

They have been operated on in every conceivable way, in every part of their course. For our purpose these operations may be divided into four groups—external shortening, shortening in the inguinal canal, intraperitoneal shortening, and transplanting.

Shortening of the ligaments after they emerge from the inguinal canal is applicable only to those cases in which there are no adhesions binding the uterus down and in which there exists no conditions of the appendages requiring surgical attention. It is a useful operation in this limited class of cases. The young and unmarried furnish a large portion of these cases, in my experience, where this operation gives satisfactory results. Shortening the ligaments in the inguinal canal seems to have no advantage over shortening them outside it, unless the peritoneal cavity is to be opened. If it is opened, the best place to do it is in the median line, one incision.

Intra-peritoneal shortening of the round ligaments has precisely the same effect on the uterus as their external shortening, but permits the operator to deal with perfect freedom with whatever complications exist. It may be done by either the abdominal or the vaginal route, as the experience or predilection of the operator dictates. In my early experience with the operation I had some relapses from folding the ligaments upon themselves, covered as they were with peritoneum and stitching them with catgut. The result was only peritoneal adhesions which were unequal to the strain put upon them by the uterus. I later obtained better results by making a slit in the peritoneum, through which I pulled the ligament and folded it upon itself to the extent necessary. After stitching it with chromicized gut, I put it back under the peritoneal cover and the slit closed. The same result could doubtless have been obtained by using silk or other permanent suture material through the peritoneum. Attaching the folded ligaments of the uterus as recommended by Dudley or Baldy seems to produce the same effect as intra-peritoneal shortening. It has been claimed—and I think justly—that when the ligaments are shortened by folding the uterine ends upon themselves their strength is increased where it least needs be.

When the round ligaments are shortened, either externally or internally, the uterus as a whole is brought downward and forward beyond its normal position. I have demonstrated this to my own satisfaction by the examining of a large number of cases operated on by myself and many by other operators. The fact that by the operation one abnormal position has been substituted for another probably accounts for some of the failures to get the anticipated relief of symptoms. The anterior displacement of the uterus can be at least partially overcome by shortening the sacro-uterine ligaments as a supplementary operation.

By causing the round ligaments to make their exit from the abdominal cavity at a point above and anterior to the inguinal canals, as well as the shortening of them, the uterus is pulled upward and forward and swung into nearly its normal position. The original Gilliam operation for this purpose has been modified for the better by Mayo, Barrett and others. It has the advantages of ventral suspension—pulls the uterus up as well as forward, acts with the greatest mechanical advantage—without its disadvantages. There is no way in which it can cause an obstruction of the bowels, nor is it likely to interfere with pregnancy or labor. I have performed this operation in most of the cases I have operated on for posterior deviation during the last two years, and with more satisfaction to myself and patients than I have obtained from any method I have previously used.

I perform the operation as follows: Make the usual incision in the median line. Deal with complications. Have an assistant catch the fascia at a point about one inch and a half above the lower end of the incision and pull it toward the opposite side; with thumb and finger seize the skin and underlying fat and retract them. With a sharp knife dissect them loose from the fascia to a point almost above the internal inguinal ring. Make a vertical incision through the fascia, take a curved clamp and with it separate the muscular fibers down to the peritoneum. With two fingers of the other hand in the abdomen the clamp is guided under the peritoneum till it passes between the folds of the broad ligament, where the round ligament is seized and pulled out through the opening in the fascia. It is securely sutured to the outer surface of the fascia with chromicized catgut and the opening closed with the same material. If the ligament has not been seized at the point which will raise the uterus to the desired level, it is easy to change it by the use of another clamp outside the opening in the fascia. Sometimes in carrying the end of the clamp over the iliac artery the peritoneum is perforated. If so, no effort is made to get under it again, but the ligament is seized, covered with the peritoneum and pulled through the opening. The peritoneum is then stripped off with dry gauze, to prevent pulling on the bladder as well as to get firm union to the ligament. The other ligament is treated in the same way, taking care that the opening through the fascia is at the same level as its fellow. I claim no originality in technic.

Of late years the importance of the sacro-uterine ligaments have been much dwelt upon, and efforts to cure retrodeviations by operation upon these cases have been made by Goff, Bovée and others, some operating through the vagina, others the abdominal incision. However important these structures may be from anatomical and physiological standpoints in health, they do not lend themselves to surgical procedures so readily as do the round ones. First, the fact that the muscular fibers in them are so few as to be almost a negligible quantity, the ligaments being little more than folds of perineum, makes them a frail tissue to trust alone to hold a uterus in position that has been displaced. Their attachment to the proximal portion of the short end of the lever, to which the uterus may be compared, places them at a great mechanical disadvantage when compared with the round ligaments, which are attached to the distal portion of the long end. For these reasons I would not depend on operation on these ligaments alone. Good results have been reported by careful men, but I do not believe sacro-uterine ligament operations will ever achieve the popularity predicted for them by Goffe.

One would expect better results from operations on the sacro-uterine ligaments in retroversion than in retroflexion, because the flexion indicates the sacro-uterine ligaments are not much relaxed, or that the uterine muscle is very deficient in tone.

Where there are no contraindications to doing so, a pessary should be worn for several weeks after operation on the round ligaments. A pessary is also of value to temporarily hold the uterus in position while involution takes place. These very valuable instruments have, from abuse and impossible things being expected of them, fallen into undeserved disrepute. They are in no sense curative agents; neither is morphine in the majority of cases in which it is used, but few would be willing to banish it from our list of therapeutic agents. Patients of mine sometimes refer to pessaries as crutches, because they permit them to be out and around when they would otherwise practically be confined to the house, if not to bed.

Almost every case in which external shortening of the round ligaments is successful could be treated satisfactorily by pessaries, but this is certainly not advisable in the young and unmarried. By the use of a pessary a patient may be made comfortable until a convenient time for her to undergo operative treatment. In the early weeks of pregnancy a pessary may hold the uterus up till it has attained sufficient size to prevent it turning backward. If a patient or her physician deems it wise to postpone radical treatment until she has borne the number of children she desires, by the use of a pessary she can do so with comparative comfort. Women suffering from retrodeviations of the uterus and nearing the menopause may choose to wear a pessary for an indefinite length of time, and wait for the senile changes in the uterus to render them of no moment rather than submit to an operation in which there is a chance of danger, or failure, slight though that chance be.

There are patients also in which organic changes in the heart or kidneys render any surgical procedure dangerous, that may be made quite comfortable by a pessary. Not every woman in which a pessary is indicated can wear one with comfort. For a pessary to be worn it is necessary that the uterus be capable of being anteverted to something more than a normal extent. If an ovary is prolapsed but goes up out of reach, when the uterus is anteverted it will not interfere with the wearing of a pessary; but if the ovary remains behind and the uterus is pushed forward, a pessary cannot be worn to any advantage. I have never been able to successfully treat with a pessary a woman the posterior cul-de-sac of whose vagina is very shallow. A good perineum is also requisite to the wearing of a pessary.

A patient wearing a pessary should be examined very frequently at first to assure the physician that it is doing all that it is expected to do and nothing more; but when it has been doing good work fcr several weeks it can very well be left alone for a few months. I have without misgivings and without ultimate regrets sent such a patient on a strenuous European tour. For purposes of cleanliness, a patient wearing a pessary should take a douche containing an alkali or a little castile soap daily.

In the third class of cases—those in which the deviation is the result of, or is accompanied by disease of the appendage so severe as to completely overshadow the deviation—the treatment is always surgical. The removal of both ovaries, which is all too often necessary in such cases, will cause the uterus to shrink up in size and make the problem of holding it in normal position easier and at the same time of less importance.

Frequently the shortening of the broad ligaments which is incident to the operation on the appendages is all that is necessary. The round ligaments may be shortened if it seems best, but the Gilliam operation or its modifications should not be done for fear of infection. Much time should not be spent in this part of the operation, for it is of minor importance, and time is a very important factor in the mortality rate in infectious cases.

It is to be hoped that evolution will finally develop the uterine supports until they are equal to their task, and that operations for posterior deviations will no longer be needed. In the meantime they can doubtless be made more rare by greater care of the health of girls at the time of puberty, and of women during labor and the puerperium.

432 West Fourth Street.

DISCUSSION.

Dr. E. Gustav Zinke: We were told a few moments ago that we all waste too much time in the discussion of papers, so

I refrain from making favorable comments upon the excellent paper just read. It is needless to say that the gentleman has covered the ground very thoroughly, not only illustrating his experience, but also his knowledge of the subject from beginning to end. One cause he left out in speaking of the conditions producing retroversion, the cause that has within the last years been a disturbing factor to railroads and traction companies when persons have met with an accident on trains and traction-cars. I refer to "falls." If the essayist did mention this it certainly escaped my attention. I know that the essayist dwelt extensively on all other causes, especially those that produce retrodeviations and prolapse of the uterus in childbearing women.

I have never seen a case in which a fall produced a uterine displacement of any kind. I also know that the textbooks speak of fall as the rarest of all causes. I have been called upon to express my opinion in three different lawsuits, and in each case I could not see that the "fall" described in each instance had anything to do with the existing displacement of the uterus. All had the typical history of uterine displacement, namely, frequent confinements and lack of care afterwards associated with laborious domestic duties. There was a history of gradual descent in each one of the cases. The opposite side, however, invariably testified that the particular accident was responsible for the displaced uterus and the diseased tubes and ovaries.

I rose to discuss the paper for the purpose of obtaining from the members of this society some better reason for retrodisplacement than a "fall" or why a "fall" should be made responsible for a displacement of the internal genitalia when the character of the accident was such that it could not produce a dislocation of these organs, and when the previous history reveals the presence of all the more frequent causes of this condition.

In one case the patient fell flat on her abdomen, and, according to the testimony on part of the plaintiff, the fall was sufficient to cause a displacement of the uterus and disease of both tubes and ovaries sufficient to make their removal necessary a few months after the accident. In my opinion the accident had nothing to do with it.

In a recent case a woman was awarded $5,000 damages for a retro-versio-flexion, prolapse of both ovaries, and lacerations of cervix and perineum, for which the accident was certainly not responsible. Does not the contradictory testimony given in these cases reflect upon the profession?

In the case last spoken of the woman had six children, the youngest of whom was at the time of the accident one or two years of age. She was brought to my office and no special reason was given for the call, except to obtain an opinion of the nature of her trouble. An examination revealed the condition just stated. She was on a traction-car which ran into a switch and was then turned upon the side. The woman slid forward in her seat and fell between it and the seat in front and upon her side. She sustained a slight contusion in the region of the right hip. She was able to get out of the car and had no trouble in going home. The doctor called the next day and found no serious injury. There was not a single symptom pointing to an acute displacement of the uterus or its adnexa. It was not until a year after that this woman was induced to sue the company for $10,000 damages.

Dr. Sigmar Stark: Dr. Zinke has brought up a very interesting subject. I do not think that the street car can be considered responsible in the case he referred to. I do not believe it possible for an injury or lifting to produce a displacement of a uterus that has otherwise not been involved in some pathological disturbance. If the woman had recently been delivered and was suffering from subinvolution, then I can understand how an accident or excessive pressure upon the anterior face of the uterus can produce displacement; but otherwise it is impossible. I likewise doubt very much that a young girl can develop a traumatic retrodisplacement if the uterus was previously normal.

In connection with the subject of the pathology of congenital retroflexion of the uterus, I wish to call attention to two conditions frequently responsible for this state that the essayist failed to refer to: First, congenital deficiency in the development of the round and the utero-sacral ligaments. I have repeatedly exposed the round ligament and found it is thin as twine and the utero-sacral ligament unrecognisable as a fold. Second, a state that has not received recognition in the textbooks, namely, an insufficient development of the cellular tissue of the vagina and parametrium.

Time and again, on examining a girl afflicted with congenital retroflexion, I have been struck by the extreme shallowness of the vagina, particularly the anterior wall. Many such cases are called prolapse of the uterus, because the cervix presents at the introitus. The next thing that strikes you in these cases is the limited mobility of the uterus and the rigidity of the vault of the vagina.

The round and sacro-uterine ligaments are practically prolongations of the musculature of the uterus. Whatever exercises an influence on the uterus will also exercise an influence on the ligaments of the uterus. The round and utero-sacral ligaments are likewise involved in the same pathological state consequent upon the causes operative in the production of subinvolution. Subinvolution of the uterus is likewise associated with a similar condition of the vagina which causes its walls to protude. This reduction of support on the part of the pelvic floor, together with the diminution of intra-abdominal support naturally consequent upon parturition, are additional factors in the establishment of uterodisplacement in the presence of subinvolution.

I wish to say a few words in regard to the features of the operation as practised by Goldspohn. I wish that I could induce some of you to take up the operation, as I look upon it as an ideal procedure. Almost any operation on the pelvic appendages can be executed at the same time, even an appendectomy, which I have frequently done in connection therewith.

I presented a paper on this subject before this society three or four years ago. At that time I advocated the employment of the pessary during the period of convalescense. Since then I have found this measure superfluous, and consequently discarded it. A further change that I have carried out is in fixation of the round ligaments as near the uterus as possible to Poupart's ligament. In some of my earliest cases I got relapses. In my last cases, during the past four years (I have done the operation over one hundred and fifty times), I have had no recurrence. One of my patients was confined just three weeks ago and attended by Dr. Wilkinson. She passed through her confinement satisfactorily. The puerperium was unaccompanied by any disturbance.

Dr. Thad. A. Reamy: Do you remove the ovarian ligament and then attach the round ligament to Poupart's ligament?

Dr. Stark: I shorten the ovarian ligament by high fixation to the uterus—for instance, when there is a prolapse of the ovary. You can do any operation on the ovary at the same time.

Dr. Reamy: Does the operation seem equal to the other or superior to it?

Dr. Stark: It is superior to it, and does not change the anatomic relations. You fasten the round ligament.

Dr. Reamy: At what point?

Dr. Stark: By three or four satures to the under surface of Poupart's ligament.

Dr. Reamy: At what place?

Dr. Stark: Through the inguinal canal.

Dr. Porter: I was very much pleased to listen to the paper. It certainly told exactly what one wanted to hear, and to me it was very interesting. It took up nearly every point of the subject. It particularly interested me because it gave weight to subinvolution as a cause of displacement. That is a matter to which I have been paying considerable attention. For a number of years I held to the opinion that whatever the position of the uterus before impregnation, it was almost certain to return to the same position. I still believe that when there is retroflexion it will practically always return. But in case of retroversion before pregnancy I now think that it is often possible to prevent a return after labor; and conversely, that faulty management of the puerperium sometimes produces retroversion in cases which originally had no displacement.

With reference to inflammation as leading to malposition, I think that this was one of the most important things mentioned in the paper. It is certainly exceedingly important from an obstetrical standpoint. That alone should urge on the obstetrician the importance of getting his patient through the puerperium without fever. An afebrile course is one of the things that prevent a displacement after labor.

In regard to operative work, I cannot say much, as my experience is limited. The best operation, in my opinion, is the Gilliam. It is the only one I have used in the few cases I have operated upon. I have been very much pleased with it. Perhaps two years ago I used it on a patient who had considerable prolapse with enlargement of the uterus and cervix. In that case there was an elongated cervix. I did an amputation of the cervix, a perineorrhaphy, and then did the Gilliam operation. There followed such complete relief that it practically changed an invalid into a woman in perfect health, enjoying every day of her life.

Dr. Reamy: Was she married?

Dr. Porter: Yes, and had borne several children.

Dr. C. D. Palmer: This subject is an extremely fertile one for discussion, but I will refer only to a few points.

I fully believe that the bad instruction, or want of instruction, given to women, in the lying-in state, is responsible for the occurrence of retroversion, when most of the cases then arise. Many women are imperfectly informed as to the judicious method of the application of the obstetric bandage, and as to the proper position for them to assume to favor normal involution of the uterus and aid the restoration of the normal position of this organ.

In my judgment, the obstetric bandage needs to be firmly applied only for the first day after parturition, then lightly applied for several weeks. She should assume the dorsal decubitus only for first few hours, and afterwards take a position on either side, somewhat prone, for two weeks at least after delivery. In perfectly normal conditions the uterus is too large and too heavy for the weakened, relaxed and stretched soft parts to do their part to sustain the subinvoluting uterus. The back wall of the uterus is usually thicker and heavier, because of the normal localisation of the placenta. This favors retroversion.

I am so impressed with the importance of this position, and this application of the obstetric bandage, that I think the period of a few weeks after delivery is a most advantageous time to seize advantage of to bring about a better position of it in all those cases in which retrodeviation has existed prior to pregnancy. And when retroversion exists it is prudent to instruct women to lie down, not on back, but on either side, and also to take once to twice every day the knee-elbow position for a half hour each time.

The bowels should always be cleared out once or more times every day, and the garments should be so adjusted as to impose the least possible weight or pressure force upon the pelvic viscera.

In reference to the other treatment of this abnormal position of the uterus, I think we could consider whether the dislocation is a movable one and a replacable one, on the one hand, or whether the organ is abnormally fixed and immovable in its awkward posture. As a rule, retroversion of a movable and replaceable uterus requires no surgical operation unless there is a torn perineum or a lacerated cervix to repair. At any rate, the abdominal cavity does not need opening, because usually there is no special disease of the uterine appendages. But if the uterus is fixed and is non-replaceable, there is always some serious affection of these appendages, and surgery may be needed to rectify the same.

Alexander's operation I have never done, and do not expect ever to do. I can do more and better without it. Of operations for this condition, I favor the opening of the abdomen and replacing mechanically the uterus, and holding it in position by the utilisation of a stout piece of cat-gut or silk first passed through the edge of one side of the incised abdominal wall, back and through the upper end of the left broad ligament beneath the point of attachment of the round ligament, then to behind the uterus, attached to it beneath its peritoneal covering; next around to the corresponding parts and places on the other side of the uterus, so that it meets and can be tied to other end of ligature in abdominal wall. This technic of procedure I prefer to Mann's, Wylie's, Gilliam's, or any other in those cases requiring abdominal section because of some complication of the ovary, tubes or pelvic peritoneum.

We would expect the cat-gut to hold firm about two weeks, the silk longer, for experience teaches us that it is absorbed not before a few months. Either ligature must be thoroughly sterilised; otherwise, the operation is dangerous. During two weeks, or longer, the patient is in bed and on her side, and before she sits up or stands a retroversion pessary is inserted, to be worn awhile.

While the utero-sacral ligaments are the most important of all to hold the uterus in its normal anteverted position, nevertheless there is no operation on it which seems to me satisfactory. For relaxed conditions of these structures I do have faith in the utilisation of the primary faradic current, used every day or so. A good, comfortably fitting pessary of the Thomas or Albert Smith pattern ought not to be overlooked for many cases of retrodeviation of the uterus.

Dr. Julia W. Carpenter: This paper is most interesting, as of all pelvic troubles retrodeviations are the most frequent. The cause and the treatment are both prolific subjects.

First, as to the cause. If one were a physician in China, and made a specialty of diseases of the feet in women, it might occur to him that the chief cause of the troubles was the deforming of the feet. Here it is the deforming of the body.

Dr. Bonifield quoted the saying that if women could go on all fours there would be no pelvic troubles. As this is impossible, why not suggest letting nature alone, as it is now, and not change the body either in position or *shape,* and then see how many pelvic troubles would disappear? If the upright position in itself is harmful, how much more so must it be when heavy weights are put upon the soft parts at the center of the trunk? The weakest part of a woman's body is the waist line. It is often so soft and flabby that the muscle fibers seem almost gone, little remaining but the skin, which can be dented inwards quite a distance. This is due to the weight and pressure of the clothing on these soft parts.

The trunk muscles are by nature among the strongest in the body, and in a man, where they are untrammeled, they furnish a large part of his great physical strength. If these muscles were weak a man could not be found strong enough to be a porter. There would be no one to lift a trunk or move furniture.

The cause of weak trunk muscles, in women, cannot be removed to any extent, as fashion is one of the most powerful of monarchs. Hence, it is in the work of physicians to relieve pelvic troubles as much as possible, while powerless to remove the chief cause.

There are many patients, however, who, when it is explained to them, will take the trouble to dress hygienically, as it can be done without changing anything in the external appearance.

Of the operation referred to, I prefer shortening the round ligaments, as that seems to give the best results.

Dr. E. Ricketts: There are cases in which retrodeviation has no symptoms of any consequence. Those cases should be let alone. In regard to the operation referred to by Dr. Bonifield—subperitoneal shortening of round ligament—I have done it in several cases with most happy results. On the other hand, the Gilliam operation will give good results.

Notwithstanding the statement made by gentlemen who have never seen a retroflexed uterus as the result of a fall or of being thrown from a horse, I have had cases of that nature. I had a case in which the uterus was retroverted and jammed. She had been thrown from a horse. The pain was simply unbearable. I gave morphine and replaced the uterus. The relief was almost instantaneous. I was called after the woman was thrown, not before, but I judge that she had not been in this condition previous to the fall.

Dr. Zinke: Did you ever see a displacement occur in which the woman was able to walk and go home after the accident?

Dr. Ricketts: In the case referred to.

Dr. E. S. McKee: I have always believed that retrodeviations can occur from falling from a horse or from a swing, or falling on the ice or from horse-back riding. Some years ago an inter-urban traction-car went off the tracks and into a stump. A woman with a young baby in her arms was thrown from her seat upon the floor with a great deal of force. She came to me afterward and I had her under observation for over a year. From the fact that she had been frequently pregnant before the accident and remained sterile afterward, and had marked retroflexion, I reasoned that she got retroflexion from the accident. A suit was brought. I gave my testimony. A distinguished specialist gave testimony directly opposite to mine. The jury gave the woman $5,000 damages, which I think she deserved.

Dr. Reamy: Shortening of the round ligaments can only have a limited range of usefulness. Within that range a proper operation is of great utility.

All know that these ligaments are not direct uterine supports. They are simply guys, preventing backward displacement of the constantly moving uterus to a degree which deprives that organ of the efficiency of its combined supports. The point at which the round ligaments leave the uterus below and in front of the Fallopian tubes, the method of entering the internal inguinal ring, passing through the inguinal canal and the external rings, is such as to allow slight gliding motion, notwithstanding loose attachments. In this motion the boundaries of the internal rings act as pullies to the ropes. The free mobility of the abdominal walls constantly changes the pulley point of bearing. Now add the considerable contractile power of the ligaments, owing to non-striated muscular fibers entering into their structure, and we have all the conditions for perfect guys. In my opinion, the attenuated and useless condition of the round ligaments observed on abdominal section in cases of chronic retroversion and prolapsus of the uterus, where the ligaments have long been in a state of tension, is largely due to the fact that contraction and relaxation have been excluded. Muscular atrophy was inevitable.

Dr. Stark informs us that his method of fixing the round ligament to Poupart's ligament is ideal—that is, I suppose that by it he effects a large per cent. of cures. I am bound to pay high respect to any utterance of Dr. Stark; he has had large experience. And yet I cannot understand how success can constantly follow a method that fixes the ligament to an immovable structure like Poupart's ligament, thus cutting off all of the essential advantages of abdominal movement. It is in violation of physical law, so manifest in this whole field.

I shall look for the method proposed by Dr. Bonifield to become popular.

Dr. Bonifield (closing): The supports of the uterus would answer their purpose admirably well if woman was a quadruped, but with her in upright position they work at a decided mechanical disadvantage, and it only takes a slight increase in weight of the uterus or a slow weakening of its supports to get it out of its position.

In regard to the uterus being suddenly thrown out of position, I had a case of a young girl of sixteen under my care whose uterus had been forced out through the vagina while in the act of depositing a heavy market basket on the floor.

I do not recall ever having seen a case of acute retroversion, but as retroversion is usually the first step in prolapse, I have no doubt but what it exists at times. The great majority of cases are chronic in character. I would be unwilling to testify in court that a displacement had, or had not, been caused by an accident, unless I had an opportunity of examining the patient before the accident occurred.

In regard to the particular operation I advise for retrodeviation, the advantages I claim for it are as follows: The abdomen is opened in the median line, giving the best opportunity for dealing with complications; the new attachments of the ligaments cause them to pull the uterus upward and forward, instead of downward and backward, as they do when they are merely shortened; the uterus is sufficiently firm, but it is not rendered immovable; the strongest part of the ligaments is utilised; pulling them under the peritoneum instead of through the peritoneal cavity is a precaution against obstruction of the bowels. The criticism that the round ligaments were never intended to support the uterus is founded on facts, but we have a pathological condition to deal with, and the histological structure of the round ligaments renders them the best support to deal with in an operative way.

It is to be borne in mind that if we would make any cure effected by operation permanent, we must improve the patient's general health and increase the tone of the uterus and its supports.

